# Editorial: Data Mining and Statistical Methods for Knowledge Discovery in Diseases Based on Multimodal Omics

**DOI:** 10.3389/fgene.2022.895796

**Published:** 2022-04-26

**Authors:** Tao Wang, Miguel E. Rentería, Jiajie Peng

**Affiliations:** ^1^ School of Computer Science, Northwestern Polytechnical University, Xi’an, China; ^2^ Key Laboratory of Big Data Storage and Management, Ministry of Industry and Information Technology, Northwestern Polytechnical University, Xi’an, China; ^3^ Department of Genetics and Computational Biology, QIMR Berghofer Medical Research Institute, Brisbane, QLD, Australia

**Keywords:** multimodal, omics, disease biology, data mining, statistical methods

Over the last decade, advances in high-throughput omics technologies and methods have enabled researchers to measure multiple biological data modalities simultaneously and accurately or to integrate multi-omics data from different sources and modalities. Numerous datasets are being rapidly generated encompassing genomics, transcriptomics, proteomics, metabolomics, phenomics, radiomics, cutting-edge 3D spatial omics, and single-cell omics data. This represents an unprecedented opportunity for knowledge discovery in disease biology, including the identification of biomarkers, functional modules, causal pathways, or regulatory networks implicated in disease, thus having also the potential to bolster current therapeutic pipelines.

In parallel, a wide-array of statistical methods have been developed to leverage availability of these data, from genome-wide association studies (GWAS) to transcription-wide association studies (TWAS), methylome-wide association studies (MWAS), molecular quantitative trait loci (molQTL) analysis, or summary-based two-sample Mendelian Randomization. However, the ability to integrate different features of existing methods is still insufficient, limiting the power for knowledge discovery. Thus, advances in data mining, or statistical and machine learning techniques are urgently needed to perform cross-modal data integration and modeling. Here, we present a Research Topic on “Data Mining and Statistical Methods for Knowledge Discovery in Diseases Based on Multimodal Omics” to showcase studies that leverage these techniques to enable discovery of disease-related knowledge and illuminate molecular mechanisms of complex diseases. After rigorous peer-review, a total of 14 outstanding articles were selected for this topic collection. Below we highlighted six of them.

Huang et al. explored the causal effects of insomnia on bipolar disorder, major depression, and schizophrenia in the European population using a two-sample Mendelian randomization approach. They first collected GWAS summary datasets for each trait and conducted meta-analyses for each trait to increase statistical power. The results of Mendelian randomization were further evaluated using extensive complementarity and sensitivity analysis. Among these psychiatric disorders, they found insomnia is causally associated with an increased risk of major depression, with an odds ratio estimated as 1.408 (95% confidence interval (CI): 1.210–1.640, *p* = 1.03E-05) in the European population. No causal association was observed for other traits. The study provides new evidence to support the causal effect of insomnia on major depression and adds to a better understanding of the relationship between sleep and psychiatric disorders.

Hamidi et al. proposed a machine learning framework to explore miRNA biomarkers and prediction for Ovarian cancer. miRNAs play an important role in cancer progression. In this study, the authors first used LASSO and Elastic Net for miRNA feature selection. They found 10 miRNA’s as potential biomarkers by comparing the expression levels in ovarian serum cancer samples and normal samples. Furthermore, they used multiple machine learning classifiers, including logistic regression, random forest, artificial neural network, XGBoost, and decision trees for ovarian cancer prediction. Experiments demonstrated the accuracy of their proposed model. The performance of the proposed models was further evaluated in external datasets.

Cerebral ischemic stroke (IS) is a complex disease caused by multiple factors, including vascular risk, genetic, and environmental factors. Identifying the genes associated with IS critical for understanding the biological mechanisms underlying the disease. Liu et al. proposed a network representation learning (NRL)-based method to identify the disease-related genes of cerebral IS. The proposed method includes three key components: capturing the topological information of the PPI network, denoising the gene feature, and optimizing a support vector machine (SVM) classifier to identify IS-related genes. The evaluation showed that the proposed method performs better than existing methods on IS-related gene prediction. In addition, the case study also shows that the proposed method can identify IS-related genes.

Recently, single-cell RNA sequencing (scRNA-seq) technology has been used to measure RNA levels at single-cell resolution to study biological functions. Xu et al. proposed an imputation method based on semi-supervised autoencoders named AdImpute. The method applies the cost function with imputation weights to learn the latent information in the data to achieve a more accurate imputation. The evaluation indicates that AdImpute is more accurate than the other four publicly available scRNA-seq imputation methods on the simulated and real data sets.

Yang et al. tackled the issue of systematic selection bias in Mendelian randomization. The authors proposed a new approach that uses control exposures based on subject-matter knowledge to triangulate the estimated causal effects vulnerable to selection bias. The proposed approach can be used to assess credible MR estimates in the presence of selection bias from selection of survivors. The authors illustrate the application of their method by validating MR estimates through a real example investigating the potential association of transferrin with stroke (including ischemic and cardioembolic stroke).

Park et al. developed an innovative approach for integrative pathway analysis that leverages genome-wide association studies summary statistics to construct genetic metabolomic scores (GMSs) that are then used as components of pathways in a hierarchical model that considers the structural relationships of SNPs, metabolites, pathways, and phenotypes. The authors applied their method to identify pathways associated with type 2 diabetes in the Korean population.

All the contributions in this special issue have been peer-reviewed by no less than two professional domain experts. We believe that the final compilation includes high-quality publications that represent significant scientific progress that will impact the relevant research communities. On this basis, we have launched a second edition of this Research Topic which is currently open for submissions.

